# Feline sporotrichosis: associations between clinical-epidemiological profiles and phenotypic-genotypic characteristics of the etiological agents in the Rio de Janeiro epizootic area

**DOI:** 10.1590/0074-02760170407

**Published:** 2018-03

**Authors:** Jéssica Sepulveda Boechat, Manoel Marques Evangelista Oliveira, Rodrigo Almeida-Paes, Isabella Dib Ferreira Gremião, Ana Caroline de Sá Machado, Raquel de Vasconcelos Carvalhaes Oliveira, Anna Barreto Fernandes Figueiredo, Vanessa Brito de Souza Rabello, Karoline Benevides de Lima Silva, Rosely Maria Zancopé-Oliveira, Tânia Maria Pacheco Schubach, Sandro Antonio Pereira

**Affiliations:** 1 Fundação Oswaldo Cruz-Fiocruz, Instituto Nacional de Infectologia Evandro Chagas, Laboratório de Pesquisa Clínica em Dermatozoonoses em Animais Domésticos, Rio de Janeiro, RJ, Brasil; 2 Fundação Oswaldo Cruz-Fiocruz, Instituto Nacional de Infectologia Evandro Chagas, Laboratório de Micologia, Rio de Janeiro, RJ, Brasil; 3 Fundação Oswaldo Cruz-Fiocruz, Instituto Nacional de Infectologia Evandro Chagas, Laboratório de Epidemiologia Clínica, Rio de Janeiro, RJ, Brasil

**Keywords:** *Sporothrix* species, sporotrichosis, cats, phenotypic characterisation, molecular characterisation

## Abstract

**BACKGROUND:**

Sporotrichosis is caused by species of the genus *Sporothrix*. From 1998 to 2015, 4,703 cats were diagnosed at the Fundação Oswaldo Cruz (Fiocruz), Rio de Janeiro, Brazil. Even after the description of the *Sporothrix* species, the characterisation of feline isolates is not performed routinely.

**OBJECTIVES:**

To characterise the clinical isolates from cats at the species level and correlate them with the clinical and epidemiological characteristics of the cats.

**METHODS:**

Forty seven *Sporothrix* spp. isolates from cats assisted at Fiocruz from 2010 to 2011 were included. Medical records were consulted to obtain the clinical and epidemiological data. The isolates were identified through their morphological and physiological characteristics. T3B polymerase chain reaction (PCR) fingerprinting was used for molecular identification of the species.

**FINDINGS:**

In phenotypic tests, 34 isolates were characterised as *S. brasiliensis*, one as *S. schenckii* and 12 as *Sporothrix* spp*.* PCR identified all isolates as *S. brasiliensis*.

**MAIN CONCLUSIONS:**

*S. brasiliensis* is the only etiological agent of feline sporotrichosis in Rio de Janeiro to date. None association was found between the isolates and the clinical and epidemiological data. In addition, we strongly recommend the use of molecular techniques for the identification of isolates of *Sporothrix* spp.

Sporotrichosis is a subcutaneous mycosis that affects humans and animals. Until few years ago, it was attributed to a single causal agent, the thermodimorphic fungus, *Sporothrix schenckii* (Lopez-Romero et al. 2011). However, from 2006, genetic variability was demonstrated among the isolates morphologically identified as *S. schenckii*, which led to the proposition of at least six species, phylogenetically described as *Sporothrix schenckii sensu stricto, Sporothrix brasiliensis*, *Sporothrix globosa, Sporothrix mexicana*, *Sporothrix pallida*, and *Sporothrix luriei* (Marimon et al. 2007, 2008). In addition, a new species called *Sporothrix chilensis* has recently been described (Rodrigues et al. 2016a).

*S. brasiliensis* has been described as an emerging species, highly pathogenic to humans and cats, which to date has a regional distribution in Brazil (Marimon et al. 2007, Arrillaga-Moncrieff et al. 2009, Oliveira et al. 2011a, Rodrigues et al. 2013b). *S. schenckii sensu stricto* is considered the second most pathogenic species (Arrillaga-Moncrieff et al. 2009), and has a worldwide distribution, with a greater preference for tropical and subtropical humid climate countries, so the highest incidence of this mycosis occurs in Americas, Africa, Australia and Asia (Marimon et al. 2007, Lopez-Romero et al. 2011, Oliveira et al. 2011a, Chakrabarti et al. 2015). The cases related to *S. globosa* occurred in humans in Europe, Asia and Americas (Marimon et al. 2007, Liu et al. 2014, Oliveira et al. 2014), including Brazil (Oliveira et al. 2011a, Rodrigues et al. 2013b). Regarding *S. mexicana*, it has been believed to be an environmental species restricted to Mexico, being isolated initially in plants from there (Marimon et al. 2007). However, this species was also isolated in a human case in Portugal (Dias et al. 2011) and in three human cases in Brazil (Rodrigues et al. 2013a). *S. luriei* is a rare species, the descriptions of human and animal sporotrichosis cases caused by this species are scarce, so far it has been reported as the causal agent of four human cases in Africa (Marimon et al. 2008) and one canine case in Brazil (Oliveira et al. 2011b). *S. pallida* is rarely described as a causal agent of sporotrichosis, with one human case described in the USA (Morrison et al. 2013) and one feline case in Brazil (Oliveira et al. 2011b). Although recently described, *S. chilensis* was isolated from a human case and environmental sample in Chile (Rodrigues et al. 2016a).

Since 1998, a large number of sporotrichosis cases in humans and cats have been described in the metropolitan region of Rio de Janeiro, Brazil, the first sporotrichosis epidemic in the form of zoonosis. Since then, the Evandro Chagas National Institute of Infectious Diseases (INI), Oswaldo Cruz Foundation (Fiocruz), a reference centre in the diagnosis of mycoses, diagnosed 4,188 human cases in the period from 1997 to 2011 (Silva et al. 2012) and 4,703 feline cases from 1998 to 2015 (Gremião et al. 2017).

Classically, the transmission of the etiological agent occurs through the skin by traumatic inoculation of the fungus present in vegetal or organic matter in the soil contaminated by conidia of *Sporothrix* spp. (Lopez-Romero et al. 2011). On rare occasions, infection can be acquired by inhalation of the conidia (Lopez-Romero et al. 2011). However, the epidemic in Rio de Janeiro has a particular profile due to the importance of zoonotic transmission, that occurs mainly through scratching and/or biting by naturally infected domestic cats (Silva et al. 2012), which transmit the yeast form of the fungus present in the nails and oral cavity of these animals (Schubach et al. 2004).

In recent years, the frequency of reported cases of sporotrichosis in cats has been increasing in Brazil, notably in Rio de Janeiro (Schubach et al. 2004, Gremião et al. 2017). Infection in cats may begin subclinically and progress to multiple skin lesions and fatal systemic form, associated or not with extracutaneous signs (Schubach et al. 2004).

A definitive diagnosis of sporotrichosis requires isolating the fungus in a culture medium (Lopez-Romero et al. 2011). The culture technique characterises the fungus as *Sporothrix* spp*.* or *S. schenkii latu sensu*, so, in order to characterise the species, polyphasic taxonomy has to be used (Oliveira et al. 2011a). The pathogenic species of the genus *Sporothrix* are very similar macroscopically; microscopically show very subtle differences and the carbohydrate assimilation pattern is very similar, which makes it difficult to identify them using only the taxonomic key initially proposed by previous study (Marimon et al. 2007).

Although there are clinical studies on feline sporotrichosis, the relationship between the clinical data of cats and the identification of the species of the genus *Sporothrix* has not been properly investigated. The aim of the present study was to characterise the clinical isolates of *Sporothrix* spp. from naturally infected cats from Rio de Janeiro, based on phenotypic and genotypic identification. In addition, the clinical-epidemiological profile of these cats and its correlation with the morphological, physiological and molecular aspects of fungal isolates were described.

## MATERIALS AND METHODS

Forty-seven isolates of *Sporothrix* spp. from cats assisted at the INI/Fiocruz from 2010 to 2011 were included, which were obtained from skin and mucosal lesions of the cats before the beginning of antifungal treatment (ketoconazole at doses 9,2 to 38,5 mg/kg/day or itraconazole at doses 8,3 to 34,5 mg/kg/day). Twenty-five cats received itraconazole (Itraconazol 100 mg; Prati Donaduzzi) as treatment and the remaining 22 cats received ketoconazole (Cetoconazol 200 mg; Prati Donaduzzi). The isolates were subsequently stored in 10% skimmed milk (Fluka Analytical, Sigma-Aldrich, Switzerland) at −20°C in the Mycology Laboratory of INI/Fiocruz. Clinical and epidemiological data were subsequently collected from the medical records of the cats.

To identify the fungal isolates at the species level, polyphasic taxonomy was used, with morphological, physiological and molecular studies, with the isolate in its filamentous form being used at all of these stages. For the phenotypic tests, the isolates were subcultured in potato dextrose agar (PDA - Difco^TM^; Becton, Dickinson and Company, Sparks MD USA) and visually examined for DHN melanin production (Almeida-Paes et al. 2015). In order to study conidiogenesis, these isolates were subcultured on corn meal agar (BBL^TM^; BD, Franklin Lakes NJ USA), and incubated at 30°C in a dark environment. After 10 days, microscopic characteristics were evaluated (Marimon et al. 2007).

The growth of colonies at 30°C and 37°C in PDA medium was evaluated after 21 days of incubation, when the diameter of the colonies was measured, with this test being performed in triplicate at different times (Marimon et al. 2007, Oliveira et al. 2011a). Carbohydrate assimilation tests were performed in 96-well racks containing Yeast Nitrogen Base - YNB culture medium (Difco™; Becton, Dickinson and Company, Sparks, MD USA) supplemented with sucrose or raffinose in a 0.5% concentration (Marimon et al. 2007, Oliveira et al. 2011a). For the control, to check the stability of the carbon sources in the culture medium, was used a *Rhodotorula mucilaginosa* isolate (IPEC41978), which has the capacity to assimilate all the carbohydrates tested. Negative control wells were supplemented with sterile distilled water and positive controls were added with glucose. In the carbohydrate assimilation test, the reading was performed on the 10th day. After this time of incubation, the media containing sucrose and raffinose were analysed. The media with similar growth to the medium with glucose were considered positive, and negative were those similar to the medium without carbohydrate. After obtaining the results of the morphological and physiological tests, we interpreted them based on the taxonomic key proposed previously (Marimon et al. 2007, 2008).

For the classification of the isolates for thermotolerance, the percentage inhibition of growth was calculated as previously reported (Almeida-Paes et al. 2015). For isolates whose growth at 37°C was reduced by 50% or more were classified as having low thermotolerance, and the isolates whose growth was reduced by less than 50% were classified as having high thermotolerance.

Subsequently, all the isolates were identified by means of genotypic testing, the genomic DNA being extracted from the filamentous form with the use of chloroform/isoamyl alcohol (24:1) (Oliveira et al. 2011a). The molecular tool used was a polymerase chain reaction (PCR) fingerprinting using the universal T3B primer (5'-AGGTCGCGGGTTCGAATCC-3') to distinguish between the species of the genus *Sporothrix* (Oliveira et al. 2015). Strains of species of the genus *Sporothrix* associated with cases found in humans and animals, *S. brasiliensis* (IPEC16490), *S. globosa* (IPEC27135), *S. mexicana* (MUM11.02), and *S. schenckii* (IPEC27722) were used as controls for molecular identification. Succinctly, we used the PCR reaction mix 10x buffer with KCl, dNTP mix 0.2 mM, MgCl_2_ 50 mM, platinum *Taq* DNA polymerase 1.0 U and 10 mM T3B primer (Oliveira et al. 2015). The T3B fingerprinting profiles obtained were analysed with Bionumerics (version 5.1; Applied Maths BVBA, Sint-Martens Latem, Belgium). Similarity coefficients were calculated using the Dice algorithm and cluster analysis was performed by means of the unweighted paired group method using arithmetic averages (UPGMA).

Georeferencing was performed from the addresses of felines, residents of the capital and other municipalities in the state of Rio de Janeiro. For this, an individual search of the addresses was done in the animal records. The address was marked as a point in the free Google Earth Pro program, allowing the identification of latitude and longitude. LatLong designed coordinates system was used. The coordinates of each point identified in Google Earth Pro were inserted in a shapefile of Rio de Janeiro, resulting in a new digital mesh. The shapefile is a vector format of geospatial data that is used in Geographic Information System (GIS) environments.

The exploratory analysis of the data was performed by applying the simple frequencies of each isolate to the species level of the genus *Sporothrix*, as well as the clinical variables of the cats (distribution of skin lesions, occurrence of lymphadenomegaly, presence of respiratory signs, presence of mucosal lesions and therapeutic response). Also, the epidemiological variables (origin and possible form of cat infection), both described in individual clinical records from the study were evaluated. For the distribution of skin lesions, the cats were divided into three groups: L1 (skin lesions in one place), L2 (skin lesions in two noncontiguous places) and L3 (skin lesions in three or more nonadjacent places) (Schubach et al. 2004).

To check the association between categorical variables, Fisher's exact test or Pearson's chi-square test were used. For the correlation of related samples the Wilcoxon test was used. For evaluation of treatment time (in weeks) to favorable outcome (clinical cure) stratified by categorical variables, the Kaplan-Meier method (KM) and Log-rank test were used. Failure events (failure of treatment, abandonment of treatment, death by sporotrichosis and death by other causes) were considered censoring by KM analysis and its length of time considered the time until last follow-up date. P-values < 0.05 indicated significant associations in the statistical tests. The data obtained with the phenotypic tests were stored and analysed in the database using the software Statistical Package for Social Science (SPSS) version 16.0.

The procedures performed in these animals were approved by the Ethics in Research Committee of the Fiocruz (CEUA/Fiocruz), Rio de Janeiro, Brazil, under license number L-041/06.

## RESULTS

Simple frequency distribution of clinical and epidemiological characteristics of the 47 cats included in this study were reported in Table I.

**TABLE I:** Distribution of the clinical and epidemiological variables of the 47 cats assisted at the Laboratory of Clinical Research on Dermatozoonosis in Domestic Animals (INI)/Fiocruz, Rio de Janeiro - Brazil, 2010 to 2011

Variables		n (%)
General health	Good	42 (89.4%)
	Regular	5 (10.6%)
Skin lesions	Present	45 (95.7%)
	Absent	2 (4.2%)
Distribution of skin lesions	L1	15 (31. 9 %)
	L2	16 (34%)
	L3	16 (34%)
Lymphadenomegaly	Present	42 (89.4%)
	Absent	5 (10.6%)
Conjunctivitis	Yes	7 (14.9 %)
	No	40 (85.1%)
Respiratory signs	Present	27 (57. 4 %)
	Absent	20 (42.6%)
Functional rhinitis	Present	20 (42.6%)
	Absent	27 (57. 4 %)
Mucosal lesion	Nasal	17 (36.2 %)
	Conjunctival	5 (10.6%)
	Absent	25 (53.2%)
Retroviruses	Positive	12 (25.5%)
(FIV/FeLV)	Negative	35 (74.5%)
Outcome	Favorable (Cure)	17 (36.2%)
	Unfavorable*	30 (63.8%)
Castrated	Yes	19 (40.4%)
	No	28 (59.6%)
Sex	Male	35 (74.5%)
	Female	12 (25.5%)

* it was considered unfavorable outcomes: failure of treatment, abandonment of treatment, death by sporotrichosis and death by other causes.

**Fig. 1:**
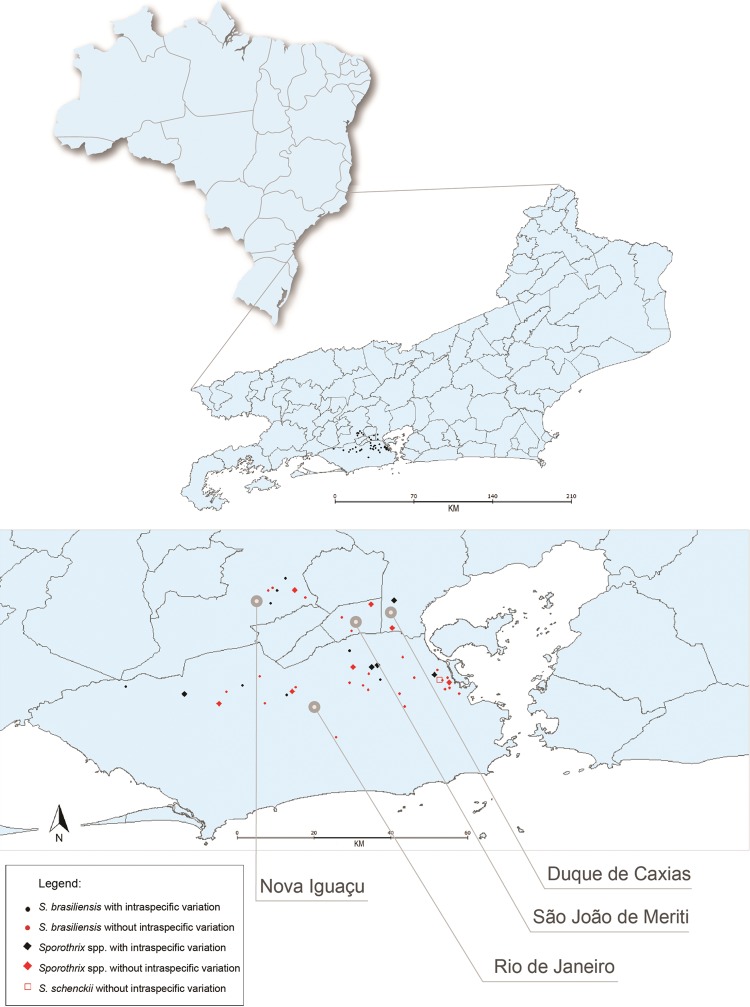
geographic distribution of 47 cases of feline sporotrichosis assisted at the Laboratory of Clinical Research on Dermatozoonosis in Domestic Animals (INI)/Fiocruz, Rio de Janeiro - Brazil, 2010 to 2011.

Thirty-five cats (74.5%) lived in the municipality of Rio de Janeiro, seven (14.9%) in Nova Iguaçu, three (6.4%) in São João de Meriti and two (4.2%) in Duque de Caxias (Fig. 1).

As to the macromorphological aspect of the fungal colonies subcultured in PDA medium, all of them had a velvety (83%) or smooth (17%) appearance, of which 41 (87.2%) had a brown (85.1%), black (2.1%) coloration with some pigment production and in six (12.7%), no pigmentation was observed in some isolates [IPEC (6G); (8G); (47G); (48G); (57G); (58G)]. With respect to the micro-morphology, all the colonies had thin hyaline, septate and ramified hyphae. Forty-six colonies (97.8%) showed hyaline and dematiaceous conidia and in one isolate (IPEC 53G) only the presence of hyaline conidia in AMC. However, macroscopically in PDA, this colony showed brown coloration, thus demonstrating melanin production by the fungus. The dematiaceous conidia had a globous or subglobous shape and the hyaline conidia were of a thin, elongated shape. No conidia with a triangular shape was found in our isolates. In the six colonies with no macroscopic pigmentation, it was possible to observe the presence of both dematiaceous conidia and hyaline conidia.

All the isolates showed excellent growth at 30°C and were thermotolerant at 37°C. The median diameter of the colonies incubated at 30°C after 21 days was 33.75 mm (20-44 mm), while the median for colonies at 37°C was 16.5 mm (5-23.5 mm). It was possible to observe that the growth of colonies at 30°C was better than at 37°C (p < 0.05).

For the classification of the isolates by thermotolerance, 31 (66%) isolates had their growth at 37°C reduced by 50% or more and were classified as having low thermotolerance. Sixteen (34%) isolates had their growth reduced by less than 50% and were classified as having high thermotolerance.

After 10 days, assimilation of glucose occurred in all isolates including the controls, 35 (72.9%) isolates did not assimilate sucrose and raffinose, 11 (22.9%) assimilated sucrose, but did not assimilate raffinose and one isolate assimilated both sugars tested. With the results of the morphological and physiological tests, it was possible to perform the phenotypic characterisation of each isolate, according to the taxonomic key previously proposed (Marimon et al. 2007). Therefore, 12 isolates (23.4%) showed no phenotypic characteristics compatible with the species of the genus *Sporothrix*, and it was not possible to define what species they were. These isolates were classified as *Sporothrix* spp. One isolate was characterised as *S. schenckii* and the remaining 34 isolates were phenotypically characterised as *S. brasiliensis* (Table II). Subsequently, they were identified using the molecular tool T3B PCR fingerprinting, and all of the control strains showed profiles with very distinct bands, which allowed us to differentiate the main species with clinical association with the species of the genus *Sporothrix*: *S. brasiliensis*, *S. globosa*, *S. mexicana* and *S. schenckii*, and it was possible to identify the feline isolates from this study at the species level. The 12 isolates phenotypically characterised as *Sporothrix* spp. obtained conclusive results using molecular identification, and were characterised as *S. brasiliensis*, as well as the isolate phenotypically characterised as *S. schenckii* (Table II).

**TABLE II:** Results of phenotypic and genotypic tests of 47 *Sporothrix* spp. isolates from cats assisted at the Laboratory of Clinical Research on Dermatozoonosis in Domestic Animals (INI)/Fiocruz, Rio de Janeiro - Brazil, 2010 to 2011

					Assimilation test		T3B fingerprinting profile compatible with *S. brasiliensis*
Phenotypic test		Dematiaceous conidia	Colony diameter larger than 50 mm	Thermotolerance	Sacarose	Rafinose	
*S. brasiliensis*	34 (72.4%)	+	No	Yes	No	No	47 (100%)
*Sporothrix* spp.	1 (2 .1%)	-	No	Yes	No	No	
	11 (2 3. 4 %)	+	No	Yes	Yes	No	
*S. schenckii*	1 (2 .1%)	+	No	Yes	Yes	Yes	

After computing the association between the clinical variables and the morpho-physiological characteristics of the isolates with a discordant phenotype, which were characterised as *Sporothrix* spp. and the isolate characterised as *S. schenckii*, there was no significant association with any of the variables tested (Table III).

**TABLE III:** Association between isolates with (Group I) and without (Group II) discordant phenotype compared to molecular characterisation, with clinical and epidemiological variations of 47 cats assisted at the Laboratory of Clinical Research on Dermatozoonosis in Domestic Animals (INI)/Fiocruz, Rio de Janeiro - Brazil, 2010 to 2011

		Group I*	Group II**	
Variables		n = 13	n = 34	p-value***
General health	Good	12 (92.3%)	30 (88.2%)	1
	Regular	1 (7.7%)	4 (11.8%)	
Distribution of skin lesions	L1	1 (7.7%)	14 (41.2 %)	0.064
	L2	7 (53.8%)	9 (26.5%)	
	L3	5 (38.5%)	11 (32.3%)	
Respiratory signs	Present	7 (53.8%)	20 (58.8%)	1
	Absent	6 (46.2%)	14 (41.2%)	
Skin lesions	Present	13 (100%)	32 (94.1%)	-
	Absent	0	2 (5.9%)	
Mucosal lesions	Present	6 (46.2%)	13 (38.2%)	0.743
	Absent	7 (53.8%)	21 (61.8%)	
Lymphadenomegaly	Present	13 (100%)	29 (85.3%)	-
	Absent	0	5 (14.7%)	
Retroviruses (FIV/FeLV)	Positive	5 (38.5%)	7 (20.6%)	0.268
	Negative	8 (61.5%)	27 (79.4%)	
Functional rhinitis	Present	7 (53.8 %)	13 (38.2%)	0.510
	Absent	6 (46.2%)	21 (61.8%)	
Outcome	Favorable (Cure)	3 (23.1%)	14 (41.2 %)	0.248
	Unfavorable	10 (76.9%)	20 (58.8%)	
Castrated	Yes	3 (23.1%)	16 (47%)	0.189
	No	10 (76.9%)	18 (53%)	
Sex	Female	11 (84.6%)	24 (70.6%)	0.464
	Male	2 (15.4%)	10 (29.4%)	

* isolates with discordant phenotype to molecular characterisation;

** isolates without discordant phenotype to molecular characterisation;

*** p < 0.05.

Even the p-value did not show statistical significance in the tests performed, the distribution between groups L1, L2 and L3 in the isolates with discordant phenotype was not proportional when compared to the distribution of lesions of the general feline population of the study.

We also computed the association between these variables and the thermotolerance results and the presence or absence of melanin for each isolate, where it was possible to observe a correlation between thermotolerance and respiratory signs and general condition of the animals (Table IV). There was no correlation with the presence or absence of visible amounts of melanin, as macroscopically visualised, with any of the variables tested (p > 0.05).

**TABLE IV:** Association between clinical variables and thermotolerance found in isolates of *Sporothrix brasiliensis* from 47 cats assisted at the Laboratory of Clinical Research on Dermatozoonosis in Domestic Animals (INI)/Fiocruz, Rio de Janeiro - Brazil, 2010 to 2011

		Low thermotolerance	High thermotolerance	
Variables		n = 31	n = 16	p-value*
General health	Good	30 (63.8%)	12 (25.5%)	0.039*
	Regular	1 (2.1%)	4 (8.5%)	
Distribution of skin lesions	L1	10 (21.3%)	5 (10.6%)	0 .175
	L2	13 (27.7%)	3 (6.4%)	
	L3	8 (17%)	8 (17%)	
Respiratory signs	Present	14 (29.8%)	13 (27.7%)	0.028*
	Absent	17 (36.2%)	3 (6.4%)	
Skin lesions	Present	30 (63.8%)	15 (31.9%)	0.626
	Absent	1 (2.1%)	1 (2.1%)	
Mucosal lesions	Present	12 (25.5%)	7 (14.9%)	0.738
	Absent	19 (40.4%)	9 (19.1%)	
Lymphadenomegaly	Present	2 8 (59.6%)	14 (29.8%)	0.766
	Absent	3 (6.4%)	2 (4.3%)	
Retroviruses (FIV/FeLV)	Positive	6 (12.8%)	6 (12.8%)	0 .176
	Negative	25 (53.2%)	10 (21.3%)	
Functional rhinitis	Present	12 (25.5%)	8 (17%)	0.458
	Absent	19 (40.4%)	8 (17%)	
Outcome	Favorable (Cure)	13 (27.7%)	4 (8.5%)	0.252
	Unfavorable	18 (38.3%)	12 (2.5%)	
Castrated	Yes	12 (25.5%)	7 (14.9%)	0.763
	No	19 (40.4%)	9 (19.1%)	
Sex	Female	8 (17%)	4 (8.5%)	1
	Male	23 (48.9%)	12 (25.5%)	

* p < 0.05.

**Fig. 2:**
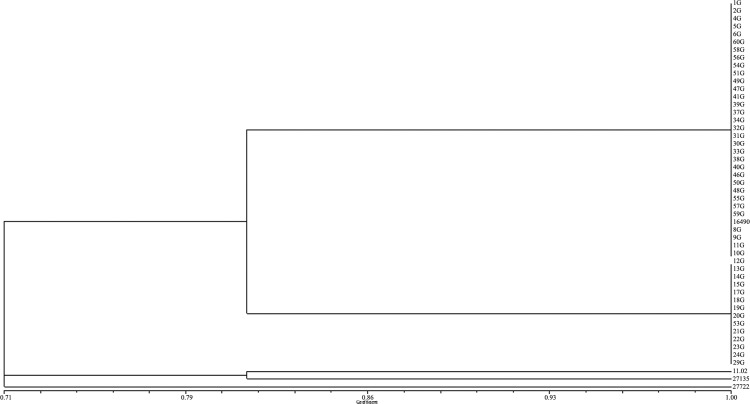
dendogram demonstrating the degree of similarity between the profiles from the T3B polymerase chain reaction fingerprinting of the 47 feline isolates characterised as *Sporothrix brasiliensis* at the Laboratory of Clinical Research on Dermatozoonosis in Domestic Animals (INI)/Fiocruz, Rio de Janeiro - Brazil, 2010 to 2011.

In the phylogenetic analysis (Fig. 2), it was possible to visualise the presence of 14 isolates (29.8%) characterised as *S. brasiliensis*, with a small intraspecific variation, that is, there was a variation when compared to the type strain of *S. brasiliensis*.

When we compare both groups with or without intra-specific variations and the clinical and epidemiological variations of the cats, it was not possible to observe any correlation (Table V).

**TABLE V:** Correlation between isolates characterised as *Sporothrix brasiliensis* that presented or not intraspecific variation, with clinical and epidemiological variations of 47 cats assisted at the Laboratory of Clinical Research on Dermatozoonosis in Domestic Animals (INI)/Fiocruz, Rio de Janeiro - Brazil, 2010 to 2011

		Group I*	Group II**	
Variables		n = 14	n = 33	p-value***
General health	Good	13 (92.9%)	29 (87.9%)	1
	Regular	1 (7.1%)	4 (12.1%)	
Distribution of skin lesions	L1	5 (35.7%)	10 (30.3%)	0.478
	L2	6 (42.9%)	10 (30.3%)	
	L3	3 (21.4%)	13 (39.4%)	
Respiratory signs	Present	10 (71.4%)	17 (51.5 %)	0.333
	Absent	4 (28.6%)	16 (48.5%)	
Skin lesions	Present	12 (85.7%)	33 (100%)	-
	Absent	2 (14.3%)	0	
Mucosal lesions	Present	7 (50%)	12 (36.4%)	0.5181
	Absent	7 (50%)	21 (63.6%)	
Lymphadenomegaly	Present	14 (100%)	28 (84.8%)	-
	Absent	0	5 (15.2%)	
Retroviruses (FIV/FeLV)	Positive	3 (21.4%)	9 (27.3%)	1
	Negative	11 (78.6%)	24 (72.7%)	
Functional rhinitis	Present	8 (57.1%)	12 (36.4%)	0. 214
	Absent	6 (42.9%)	21 (63.6%)	
Outcome	Favorable (Cure)	6 (42.9%)	11 (33.3%)	0.740
	Unfavorable	8 (57.1%)	22 (66.7%)	
Castrated	Yes	7 (50%)	12 (36.4%)	0.518
	No	7 (50%)	21 (63.4%)	
Sex	Female	4 (28.6%)	8 (24.2%)	0.731
	Male	10 (71.4%)	25 (75.8%)	

* isolates with intraspecific variation in molecular technique;

** isolates without intraspecific variation in molecular technique;

*** p < 0.05.

When we observed the distribution of isolates with discordant and concordant phenotype and isolates with and without intraspecific variation, by the municipalities of Rio de Janeiro state, it was possible to observe that isolates characterised phenotypically as *S. brasiliensis* and with intraspecific variation are not present in the municipalities of Duque de Caxias and São João de Meriti. Isolates characterised as *Sporothrix* spp. and with intraspecific variation were not found only in São João de Meriti (Fig. 1).

**Fig. 3:**
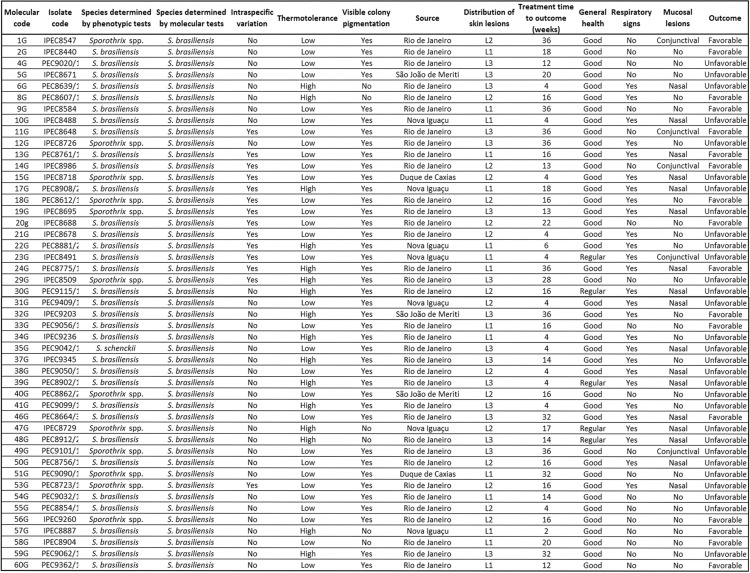
clinical, epidemiological and phenotypic and molecular characterisation of the 47 isolates of the cats assisted at the Laboratory of Clinical Research on Dermatozoonosis in Domestic Animals (INI)/Fiocruz, Rio de Janeiro - Brazil, 2010 to 2011.

In Fig. 3 it is possible to observe the 13 animals that showed a discordant phenotype through molecular characterisation and the 14 animals that within the molecular characterisation showed intraspecific variations, together with the clinical and epidemiological characteristics of each animal.

When we described the data of the 14 strains that showed intraspecific variations, 10 were from cats that showed respiratory signs, seven from those showing mucosal lesions, eight from cats with unfavorable outcomes and all of them produced melanin. In the 13 strains that showed phenotypes classified as *Sporothrix* spp. and *S. schenckii*, six of these animals had mucosal lesions, seven with the presence of respiratory signs, 10 with unfavorable outcomes and all 13 isolates had visible pigmentation.

In the 47 animals, 17 cats obtained clinical cure, of these, 11 were treated with itraconazole and six with ketoconazole. Unfavorable outcome occurred in 30 cats, of these, 14 were treated with itraconazole and 16 with ketoconazole. Comparing the treatment time to favorable outcome in groups by Kaplan-Meier method, with and without intraspecific genetic variation, no significant difference was observed (Log rank p-value = 0.55).

**Fig. 4:**
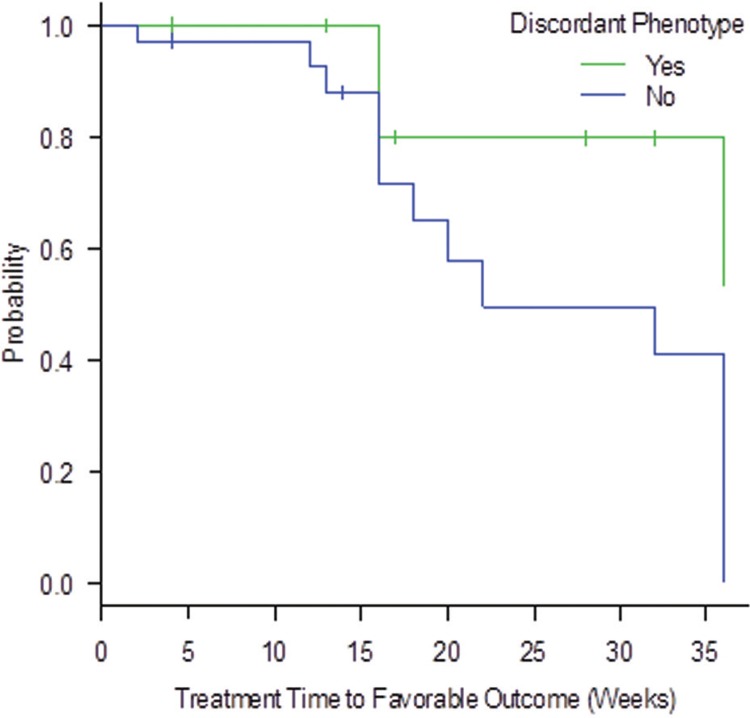
association between groups with and without discordant phenotype compared to molecular characterisation, and the treatment time to favorable outcome (in weeks) of cats assisted at the Laboratory of Clinical Research on Dermatozoonosis in Domestic Animals (INI)/Fiocruz, Rio de Janeiro - Brazil, 2010 to 2011.

In the 13 strains that showed discordant phenotypes, only three had a cure as the clinical outcome, and the median time of treatment until cure was 31 weeks. The remaining 14 animals, without discordant phenotype and which also had a cure as the outcome, obtained a median time of treatment of 23 weeks. Thus, comparing the treatment time to favorable outcome with the phenotype, the group with discordant phenotype had a longer time to reach a favorable outcome when compared to the group without discordant phenotype (p-value = 0.041), and it is possible to observe in the Fig. 4.

## DISCUSSION

The main focus of the present study was the phenotypic and molecular characterisation at the species level of 47 isolates of *Sporothrix* spp. from cats treated at INI/Fiocruz, Rio de Janeiro, Brazil, from 2010 to 2011. Polyphasic taxonomy was performed to identify the isolates. The clinical and epidemiological characteristics of the feline population from which the isolates were obtained were included in the study and analysed together with the results of the characterisation of the fungal species.

After the description of the species of the genus *Sporothrix*, the identification of clinical isolates has been performed worldwide, especially in regions where a large number of sporotrichosis cases occur (Marimon et al. 2008, Oliveira et al. 2011a, Almeida-Paes et al. 2014, Liu et al. 2014, Rodrigues et al. 2016b). However, the number of isolates characterised from animals is still low in relation to isolates obtained from humans (Oliveira et al. 2011b, Rodrigues et al. 2013b), mainly to the fact that zoonotic sporotrichosis is not cosmopolitan as classical sporotrichosis. In addition, the association between clinical/epidemiological data of the cats with sporotrichosis and *S. brasiliensis* has not been performed so far. Cases of feline sporotrichosis have been documented in Malaysia, and studies conducted the characterisation of these isolates. However, the clinical and epidemiological characteristics of the cats have been poorly explored (Kano et al. 2015, Han et al. 2017).

The description of the clinical profile of the feline population affected by sporotrichosis in Brazil and in other countries is similar (Davies & Troy 1996), even though the epidemic occurring in Rio de Janeiro has particularities such as zoonotic transmission. In our study, it was uncastrated males, without any defined breed, and with unrestricted access to the street that were the most frequently affected by sporotrichosis, corroborating with the findings of other authors who carried out studies in Brazil, in the same region of the epidemic and in other places (Schubach et al. 2004, Pereira et al. 2010, Madrid et al. 2012, Gremião et al. 2015).

The animals from which the isolates were obtained came from the state of Rio de Janeiro, the vast majority from the municipality of Rio de Janeiro (74.5%), followed by Nova Iguaçu (14.9%), São João de Meriti (6.4%), and Duque de Caxias (4.2%). It was reported (Silva et al. 2012) that the human cases of sporotrichosis in the metropolitan region of Rio de Janeiro were located in greater concentrations in the city of Rio de Janeiro, followed by neighboring municipalities, such as Duque de Caxias, São João de Meriti, Nova Iguaçu, Nilópolis, Belford Roxo and Mesquita. The fact that there are more cases, both human and animal, in Rio de Janeiro is problaby due to the fact that Fiocruz is based in the same municipality. These findings corroborate the epidemiological data found in our study, since the animals included were mostly from the municipality of Rio de Janeiro and the municipalities of the Baixada Fluminense region, with the areas where there were human and animal overlapping cases.

Nodules and skin ulcers were the most commonly observed lesions in the cats evaluated, corroborating the findings of other authors (Davies & Troy 1996, Schubach et al. 2004, Madrid et al. 2010, Pereira et al. 2010). However, the distribution of skin lesions observed in our study corroborated findings from the two largest studies on feline sporotrichosis performed to date (Schubach et al. 2004, Pereira et al. 2010), with a higher frequency of L3 group followed by L1 and L2 groups.

The extracutaneous signs were similar to those previously described (Pereira et al. 2010), with sneezing, rhinorrhea and lymphadenomegaly the most frequent ones. The presence of respiratory signs was associated with therapeutic failure and the death of the animals (Pereira et al. 2010). The difficulty in treating cats with sporotrichosis when there is nasal involvement, indicating that the severity and extent of the lesions in this region may make it difficult to cure, has been demonstrated previously (Gremião et al. 2015).

The cats studied here were submitted to systemic oral azole therapy and the occurrence of unfavorable outcomes was striking. The treatment of feline sporotrichosis with azole is complicated in some cases, with clinical cure rates varying in studies conducted in Rio de Janeiro (Schubach et al. 2004, Pereira et al. 2010, Gremião et al. 2015), and this may be related to the etiological agent predominantly associated with the epizotic disease in the region, *S. brasiliensis*, which is the most virulent of the pathogenic species of genus *Sporothrix* (Arrillaga-Moncrieff et al. 2009, Almeida-Paes et al. 2015), in murine model studies. However in Rio de Janeiro epidemics, Almeida-Paes et al. (2014) reported that human sporotrichosis caused by *S. schenckii* requires extended treatment times and high doses of itraconazole when compared to cases of sporotrichosis caused by *S. brasiliensis*. This may suggest that *Sporothrix* virulence may be associated with the initial severity of the cases.

The characterisation of the isolates of *Sporothrix* spp. from cats is fundamental in regions where zoonotic transmission occurs, because these animals are the main source of infection of this fungus for humans in most cases (Oliveira et al. 2011a, Gremião et al. 2017).

However, up to now, characterisation at the species level proposed by Marimon et al. (2007) is not performed as routine in most diagnostic laboratories because it requires specialised training for personnel, adequate laboratory structure and these techniques are time-consuming.

Despite the importance of knowing which pathogenic species of the genus *Sporothrix* are circulating in Brazil, especially in the regions where the largest number of feline sporotrichosis occur, few studies on isolates obtained from cats have been carried out to identify species circulating in the state of Rio de Janeiro, the main area of zoonotic transmission by the genus *Sporothrix*. Since the beginning of the sporotrichosis epidemic in Rio de Janeiro in 1998, 15 feline isolates from this region (Rodrigues et al. 2013b) were characterised by means of molecular analysis, however, this was limited to the identification the species, without taking into account the clinical and epidemiological characteristics of the feline population as described in the present study.

*S. brasiliensis* has been described as an emerging species, highly pathogenic for humans and animals, but with a regional geographic distribution associated with Brazil (Marimon et al. 2007, Arrillaga-Moncrieff et al. 2009, Oliveira et al. 2011a, Rodrigues et al. 2013b). This was the only species found in our study, corroborating with the single study with isolates from the epizootic region of feline sporotrichosis in Rio de Janeiro (Rodrigues et al. 2013b). In the southeastern region of Brazil, authors have described the prevalence of *S. brasiliensis* in cats affected by sporotrichosis, and reported cases in all the states of Rio de Janeiro (Rodrigues et al. 2013b), São Paulo (Rodrigues et al. 2013b), Minas Gerais (Rodrigues et al. 2013b) and Espírito Santo (Oliveira et al. 2013), corroborating our results. We believe that these findings are basically due to the proximity of the states of São Paulo, Minas Gerais and Espírito Santo to the epizootic region of Rio de Janeiro and the possibility of cats migrating from the endemic area to these adjacent regions with their owners. Outside the Southeast region, in the state of Rio Grande do Sul, there has also been a description of *S. brasiliensis* circulating among animals (Oliveira et al. 2011b, Sanchotene et al. 2015).

In six isolates characterised by molecular techniques as *S. brasiliensis*, the pigmentation of the colony was not observed macroscopically, but they developed globular and dematiaceous conidia, as described in previous studies (Oliveira et al. 2011a). In one isolate, although macroscopically was observed pigmentation, microscopically there was only production of hyaline conidia. The major explanation for this variation are the different culture media recommended for macroscopic and microscopic evaluation of *Sporothrix* spp.

In 12 isolates (25.5%) it was not possible to reach a taxonomic classification at the species level only by means of phenotypic characteristics, other authors also failed to reach a taxonomic classification (Oliveira et al. 2011a, 2013). In 11 of these isolates, only sucrose was assimilated in the carbohydrate assimilation test, this being suggestive of the species *S. pallida* or *S. globosa*, but these are not classified as *S. pallida* because of the presence of dematiaceous conidia and neither as *S. globosa* because these isolates are thermotolerant at 37°C, which do not occur with *S. globosa* isolates, that are unable to grown at this temperature (Marimon et al. 2007). In one isolate, sucrose and raffinose was not assimilated in the carbohydrate assimilation test, which is suggestive of *S. brasiliensis*, but there was no development of dematiaceous conidia. Therefore, it was not possible to identify the species of these 12 isolates by this method. One isolate was phenotypically identified as *S. schenckii*, but did not showed triangular conidia, which was also observed in a previous study (Oliveira et al. 2011a). According to Marimon et al. (2007), the triangular conidia could be a peculiar feature of the strains characterised as *S. schenckii*. Since this isolate was further characterised as *S. brasiliensis*, the absence of triangular conidia corroborates the study of Marimon et al. (2007). Due to this diversity of results in the phenotypic tests, we strongly suggest that the identification of species of the genus *Sporothrix* using only morphological and physiological tests should be used with precaution.

All isolates of this study showed growth at 37°C, and they were classified into those having low and high thermo-tolerance. These isolates showed association with respiratory signs and general health condition, probably due to the fact that strains with higher thermotolerance have greater ease of dissemination (Almeida-Paes et al. 2015), generating more severe conditions and respiratory signs in the cats.

All isolates included in this study were identified as *S. brasiliensis* by the T3B PCR fingerprinting technique. Thus, we detected 13 isolates with differences between their phenotypic and molecular identification, corroborating reports from other authors studying *Sporothrix* isolates from humans in the same region (Oliveira et al. 2011a, Almeida-Paes et al. 2014) and in different regions (Rodrigues et al. 2013a).

Despite the inconclusive phenotypic results in 12 isolates, the characterisation was performed using the molecular T3B PCR fingerprinting technique, through which we successfully identified the species *S. brasiliensis* in all of these cases. In another case it was categorised phenotypically as *S. schenckii*, however, in the molecular technique it was characterised as *S. brasiliensis*. Additionally, this technique proved to be simple and fast, as previously demonstrated by our group (Oliveira et al. 2015). These results suggest that the characterisation of pathogenic species of the genus *Sporothrix* should be based on the association of phenotypic and molecular techniques that allow one to differentiate between the species.

There was a small degree of intraspecific variation among our isolates classified as *S. brasiliensis*, as has also been found in other study with human isolates (Oliveira et al. 2015). However, no association was found between these findings and the clinical and epidemiological data from the felines, suggesting that more studies should be carried out with a larger number of isolates and more techniques, in order to study possible relationships between the genotypes and phenotypes of *Sporothrix* spp. and the clinical characteristics of the animals naturally infected by these fungi.

The 13 isolates phenotypically characterised that had discordant results compared to molecular results, when associated with treatment time to favorable outcome showed a significant result, demonstrating that these animals had a longer time to reach the cure, when compared to the group that did not had discordant phenotype. Although the p-value was not statistically significant, the distribution between the L1, L2 and L3 groups in the isolates that showed phenotype discordant from the molecular identification, it was not proportional to the distribution of lesions in the general feline population in the study. The major inconsistency in the phenotypic tests was the assimilation of unexpected carbohydrates, which can reflect an adaptation to survive under unpredicted nutritional conditions. We suggest therefore, that isolates that show a discordant phenotype may be more virulent, requiring more investigations on virulence so that such a comparison can be made.

We conclude that *S. brasiliensis* is the etiological agent associated with feline sporotrichosis in the metropolitan region of Rio de Janeiro, Brazil. For now, we recommend the use of molecular techniques for the effective identification of feline isolates, due to the lack of agreement between phenotypic and genotypic methods. Although the number of isolates of *Sporothrix* from cats with sporotrichosis included in this study is the largest so far in Brazil, it was still not representative due to the large number of cats presenting this mycosis in this country, with the sample number being a limiting factor for some of the analyses performed. In order for the correlation between the species found and the clinical and epidemiological data on the cats might be performed more reliably and so that an investigation into other species circulating in the region might be carried out, we suggest performing a multi-center study that includes a larger quantity of clinical isolates of *Sporothrix* in different areas of high occurrence of the disease.
